# The angiopoietin-like protein 4, apolipoprotein C3, and lipoprotein lipase axis is disrupted in patients with rheumatoid arthritis

**DOI:** 10.1186/s13075-022-02784-z

**Published:** 2022-04-29

**Authors:** Laura de Armas-Rillo, Juan Carlos Quevedo-Abeledo, Vanesa Hernández-Hernández, Antonia de Vera-González, Alejandra González-Delgado, José A. García-Dopico, Miguel Á. González-Gay, Iván Ferraz-Amaro

**Affiliations:** 1 Division of Health Sciences, Universidad Europea de Canarias, Tenerife, Spain; 2 Division of Rheumatology, Hospital Doctor Negrín, Las Palmas de Gran Canaria, Spain; 3 Division of Rheumatology, Hospital Universitario de Canarias, Tenerife, Spain; 4 Division of Central Laboratory, Hospital Universitario de Canarias, Tenerife, Spain; 5 Epidemiology, Genetics and Atherosclerosis Research Group on Systemic Inflammatory Diseases, Hospital Universitario Marqués de Valdecilla, IDIVAL, Santander, Spain; 6 Division of Rheumatology, Hospital Universitario Marqués de Valdecilla, Universidad de Cantabria, Santander, Spain; 7 Cardiovascular Pathophysiology and Genomics Research Unit, School of Physiology, Faculty of Health Sciences, University of the Witwatersrand, Johannesburg, South Africa

**Keywords:** Rheumatoid arthritis, Dyslipidemia, Inflammation, Lipoprotein lipase, Angiopoietin-like protein 4, Apolipoprotein C3

## Abstract

**Background:**

Modulators of triglyceride metabolism include lipoprotein lipase (LPL), angiopoietin-like protein 4 (ANGPTL4), and apolipoprotein C-3 (ApoC3). There is evidence on the influence of this triangle of molecules on an increased risk of atherosclerotic cardiovascular disease (CV) in the general population. Patients with rheumatoid arthritis (RA) present changes in lipid profiles and accelerated CV disease. In the present study, we set out to study whether the ANGPTL4, ApoC3, and LPL axis differs in subjects with RA compared to controls. In a further step, we investigated the relationship of this axis with subclinical atherosclerosis in patients with RA.

**Methods:**

Cross-sectional study that included 569 individuals, 323 patients with RA and 246 age-matched controls. ANGPTL4, ApoC3 and LPL, and standard lipid profiles were analyzed in patients and controls. Carotid intima-media thickness (cIMT) and carotid plaques were assessed in RA patients. A multivariable analysis was performed to assess whether the ANGPTL4, ApoC3, and LPL axis was altered in RA and to study its relationship with RA dyslipidemia and subclinical carotid atherosclerosis.

**Results:**

Most lipid profile molecules did not differ between patients and controls. Despite this, and after fully multivariable analysis including CV risk factors, use of statins, and changes in the lipid profile caused by the disease itself, patients with RA showed higher serum levels of ANGPTL4 (beta coef. 295 [95% CI 213–376] ng/ml, *p*<0.001) and ApoC3 (beta coef. 2.9 [95% CI 1.7–4.0] mg/dl, *p*<0.001), but lower circulating LPL (beta coef. −174 [95% CI −213 to −135] ng/ml, *p*<0.001). ANGPTL4 serum levels were positively and independently associated with a higher cIMT in patients with RA after fully multivariable adjustment.

**Conclusion:**

The axis consisting in ANGPTL4, ApoC3, and LPL is disrupted in patients with RA. ANGPTL4 serum levels are positively and independently associated with a higher cIMT in RA patients.

## Background

Key molecules involved in the metabolism of triglycerides include lipoprotein lipase (LPL), angiopoietin-like protein 4 (ANGPTL4), and apolipoprotein C-3 (ApoC3). LPL is the primary enzyme that hydrolyzes lipoprotein triglycerides releasing free fatty acids for utilization by and clearance from tissues [1]. Among the endogenous modulators of LPL are ANGPTL4 that inhibits LPL and modulates the uptake of free fatty acids in fasting and fed states [2] and ApoC3 that also inhibits the lipolysis of triglyceride-rich lipoproteins by LPL [3].

There is evidence on the influence of this axis constituted by ANGPTL4-LPL-ApoC3 on an increased risk of atherosclerotic cardiovascular disease (CV) in the general population. It has emerged not only from genetic studies, but also from the relationship of circulating levels of these molecules with CV disease and CV events. For example, loss-of-function mutations in APOC3 were associated with low levels of triglycerides and a reduced risk of ischemic CV disease, and elevated ApoC3 levels are associated with increased triglyceride levels and elevated risk of atherosclerotic CV disease [4]. Besides, truncating mutations that increase LPL activity decrease serum triglyceride levels and the risk of CV disease, whereas mutations that diminish LPL function have been shown to increase serum triglyceride levels [5]. Similarly, carriers of inactivating mutations in ANGPTL4 exhibit lower triglyceride levels and a lower risk of coronary artery disease than non-carriers [6].

Rheumatoid arthritis (RA) has been associated with a modified lipid profile that is considered a consequence of the inflammatory state that accompanies the disease [7]. RA is also known to be associated with a higher incidence of CV events [8]. In the present study, we set out to study whether the ANGPTL4, ApoC3, and LPL axis differs in subjects with RA compared to controls. In a further step, we investigated the relationship of this axis with subclinical atherosclerosis in patients with RA.

## Material and methods

### Study participants

This was a cross-sectional study that included 569 individuals, 323 patients with RA and 246 age-matched controls. All RA patients were 18 years old or older and fulfilled the 2010 ACR/EULAR diagnostic criteria [9]. They had been diagnosed by rheumatologists and were periodically followed up at rheumatology outpatient clinics. For the purposes of inclusion in the present study, the duration of RA disease was required to be ≥1 year. Although anti-tumor necrosis factor-alpha (TNF) treatment has been associated with changes in lipid profiles [10], RA patients undergoing TNF-alpha antagonists or other biologic therapies were not excluded from the present study. Apart from possible statin use, age-matched controls included in the study were required not to have conditions or drug treatment that could influence lipids and were not taking any other lipid-lowering medications. The controls were community-based, recruited by general practitioners in primary health centers. Moreover, controls with a history of any inflammatory rheumatic diseases were excluded, as well as those with a history of CV disease. None of the controls was receiving glucocorticoids. However, since they are often used in the management of RA, patients taking prednisone, or an equivalent dose ≤10 mg/day, were not excluded. As previously mentioned, both patients and controls under statin treatment were allowed to participate in the study. Patients and controls were excluded if they had a history of myocardial infarction, angina, stroke, a glomerular filtration rate <60 ml/min/1.73 m^2^, a history of cancer, or any other chronic disease, or evidence of active infection. The study protocol was approved by the Institutional Review Committee at Hospital Universitario de Canarias and at Hospital Universitario Doctor Negrín (both in Spain), and all subjects provided informed written consent.

### Data collection and laboratory assessments

Individuals included in the study completed a CV risk factor and medication use questionnaire and underwent a physical examination. Weight, height, body mass index, abdominal circumference, and systolic and diastolic blood pressure (measured with the participant in a supine position) were assessed under standardized conditions. Information regarding smoking status (current smoker versus non-smoker) and hypertension was obtained from the questionnaire. Medical records were reviewed to ascertain specific diagnoses and medications. Disease activity in patients with RA was measured using the Disease Activity Score (DAS28) in 28 joints [11], the Clinical Disease Activity Index (CDAI) [12], and the Simple Disease Activity Index (SDAI) [13]. Disease disability was measured through the Health Assessment Questionnaire (HAQ) score [14].

Serum LPL mass was measured using a sensitive sandwich enzyme-linked immunosorbent assay (ELISA) (Biomatik, Cambridge, Canada). The assay sensitivity (minimum detectable concentration) for LPL was 0.58 ng/ml. Precision was estimated as an inter-assay <15% and an intra-assay <10% coefficients of variability. ANGPTL4 was assessed through R&D Duoset ELISA (Abingdon, UK). ANGPTL4 minimum detectable values were 1.3 ng/ml and both inter- and inter-assay coefficients of variability were <10%. For the detection of ApoC3, an ELISA kit was used (Elabscience, USA). No significant cross-reactivity or interference between human ApoC3 and analogues is observed with this kit. Both intra- and inter-coefficients of variability are < 10% for this assay. Cholesterol, triglycerides, and HDL cholesterol were measured using the enzymatic colorimetric assay. LDL cholesterol was calculated using the Friedewald formula. A standard technique was used to measure the erythrocyte sedimentation rate (ESR) and high-sensitivity C-reactive protein (CRP).

### Carotid ultrasound assessment

Carotid ultrasound examination was used to assess cIMT in the common carotid artery and to detect focal plaques in the extracranial carotid tree in patients with RA [15]. A commercially available scanner, the Esaote Mylab 70 (Genoa, Italy), equipped with a 7–12-MHz linear transducer and an automated software-guided radiofrequency technique, Quality Intima Media Thickness in real-time (QIMT, Esaote, Maastricht, Holland), was used for this purpose. As previously reported [15], based on the Mannheim consensus, plaque criteria in the accessible extracranial carotid tree (common carotid artery, bulb and internal carotid artery) were defined as follows: a focal protrusion in the lumen measuring at least cIMT >1.5 mm, a protrusion at least 50% greater than the surrounding cIMT, or arterial lumen encroaching >0.5 mm [16].

### Statistical analysis

Demographic and clinical characteristics in patients with RA and controls were described as mean (standard deviation) or percentages for categorical variables. For non-normally distributed continuous variables, data were expressed as median and interquartile range (IQR). Univariable differences between patients and controls were assessed through the Student *T*, Mann–Whitney *U*, chi-square, or Fisher exact tests according to normal distribution or number of subjects. Differences between patients and controls regarding their lipid profiles were assessed through multivariable regression analysis. Confounding variables in this analysis were those with a statistical *p* value <0.20 for those differences in traditional CV risk factors between patients and controls. To neutralize the effect of other modifications on the lipid profile, an additional multivariable analysis was constructed, adding to the model those differences in lipid-related molecules between patients and controls with a *p* value <0.20. Demographic- and disease-related data associations with ANGPTL4, LPL, and ApoC3 are shown using univariable linear regression. Mediation analysis [17] was used to further understand the associations of RA with changes in ANGPTL4, LPL, and ApoC3. Therefore, an attempt was made to assess whether any of these molecules was responsible for the change of the others. Therefore, in these significant relationships, it was ruled out that there was no mediation of another molecule. In cases where the mediation was not significant, it was established that the relationship is direct and not mediated by another molecule. Mediation analysis estimated two models as previously described [18]: a model for the mediator conditional on exposure and covariates, and another model for the outcome conditional on exposure, the mediator and covariates. All the analyses used a 5% two-sided significance level and were performed using SPSS software, version 25 (IBM, Armonk, NY, USA), and Stata software, version 17/SE (StataCorp, College Station, TX, USA). *p* values <0.05 were considered statistically significant.

## Results

### Demographic and disease-related data

A total of 569 participants, 323 patients with RA and 246 controls, were included in this study. Demographic- and disease-related characteristics of the participants are shown in Table 1. Patients and controls showed no differences in age (54 ± 16 vs. 55 ± 10 years, *p*=0.62) nor in the frequency of the CV risk factors smoking, obesity, hypertension, and type 2 diabetes mellitus. Similarly, the use of statins did not differ between patients and controls (32 vs. 27%, *p*=0.19). Contrary, patients with RA were more frequently female and had a lower BMI and abdominal circumference. However, for these differences, the size effect was found to be small.

**Table 1:** Demographics, cardiovascular risk factors, and disease-related data in subjects

	Controls	RA	
	(*n*=246)	(*n*=323)	*p*
Age, years	54 ± 16	55 ± 10	0.62
Female, *n* (%)	162 (66)	263 (81)	**<0.001**
BMI, kg/m^2^	30 ± 3	28 ± 5	**<0.001**
Abdominal circumference, cm	100 ± 6	97 ± 13	**<0.001**
Cardiovascular data			
CV risk factors, *n* (%)			
Current smoker	47 (19)	64 (20)	0.83
Obesity	67 (27)	104 (32)	0.20
Hypertension	92 (37)	101 (31)	0.13
Diabetes mellitus	42 (17)	42 (13)	0.16
Blood pressure, mm Hg			
Systolic	139 ± 8	133 ± 19	**<0.001**
Diastolic	84 ± 5	81 ± 12	**0.001**
Statins, *n* (%)	66 (27)	104 (32)	0.19
Disease-related data			
CRP at time of study, mg/l	2.0 (1.1–4.6)	2.6 (1.3–6.1)	**0.023**
Disease duration, years		8 (4–15)	
ESR at time of study, mm/1° hour		25 (12–45)	
Rheumatoid factor, *n* (%)		218 (67)	
ACPA, *n* (%)		179 (55)	
DAS28-ESR		2.32 ± 1.19	
DAS28-PCR		2.54 ± 1.05	
SDAI		12 (6–20)	
CDAI		8 (4–14)	
HAQ		0.750 (0.250–1.250)	
Current drugs, *n* (%)			
Prednisone		123 (38)	
Prednisone doses, mg/day		5 (3–5)	
NSAIDs		154 (48)	
DMARDs		277 (86)	
Methotrexate		238 (74)	
Leflunomide		72 (22)	
Hydroxychloroquine		37 (11)	
Sulfasalazine		25 (8)	
Anti-TNF therapy		68 (21)	
Tocilizumab		17 (5)	
Rituximab		6 (2)	
Abatacept		6 (2)	
JAK inhibitors		7 (2)	
Historical disease-related data			
History of extraarticular manifestations, *n* (%)		24 (7)	
Erosions, *n* (%)		113 (35)	
CRP at time of disease diagnosis, mg/l		6.4 (2.5–17.1)	
CRP >3 at time of disease diagnosis, *n* (%)		152 (47)	
ESR at disease diagnosis, mm/1° hour		31 ± 19	
Subclinical atherosclerosis			
Carotid IMT, microns		698 ± 137	
Carotid plaques, *n* (%)		124 (38)	

Data represent means ± SD or median (IQR) when data were not normally distributed

*CV* cardiovascular, *LDL* low-density lipoprotein, *HDL* high-density lipoprotein, *CRP* C-reactive protein, *NSAID* nonsteroidal anti-inflammatory drugs, *DMARD* disease-modifying antirheumatic drug, *TNF* tumor necrosis factor, Obesity, *ESR* erythrocyte sedimentation rate, *BMI* body mass index, *DAS28* Disease Activity Score in 28 joints, *ACPA* anti-citrullinated protein antibodies, *CDAI* Clinical Disease Activity Index, *SDAI* Simple Disease Activity Index, *HAQ* Health Assessment Questionnaire

The median duration of the disease in RA patients was 8 (IQR 4–15) years. Sixty-seven percent of patients were positive for rheumatoid factor and 55% for ACPA. Disease activity measured by DAS28-ESR showed a value of 2.32 ± 1.19. Thirty-eight percent of the patients were being treated with prednisone and 86% were taking at least one conventional DMARD in any of its types, being methotrexate the most widely used (74%). The frequency of use of other treatments is shown in Table 1. Additionally, the mean values of CRP and ESR at the time of the study were respectively 2.6 (IQR 1.3–6.1) mg/l and 25 (IQR 12–45) mm/1st hour. The cIMT of patients with RA was 698 ± 137, and 38% of these presented carotid plaque on ultrasound examination. Additional information on patients and controls is shown in Table 1.

### Multivariable analysis of the differences in lipid profiles between RA patients and controls

In general, lipid profile did not differ between RA patients and controls in the univariable analysis. Only HDL cholesterol was found to be significantly higher in RA patients compared to controls (56 ± 15 mg/dl vs. 52 ± 15 mg/dl, *p*=0.001). Despite this, ANGPTL4, ApoC3, and LPL were found to be different in patients with RA compared to controls. In this sense, in the univariable analysis, ANGPTL4 (151 [IQR 90–290] ng/ml vs. 73 [IQR 47–121] ng/ml, *p*<0.001) and ApoC3 (8.8 ± 5.2 mg/dl vs. 6.2 ± 5.6, *p*<0.001) were found to be significantly higher in patients with RA. Contrary, in this univariable analysis, LPL was found to be lower in RA when compared to controls (99 [IQR 60–156] ng/ml vs. 230 [IQR 183–328] ng/ml, *p*<0.001).

In the full adjustment model (model 1 in Table 2), most of these differences between the two populations were maintained with some exceptions. In this sense, apolipoprotein A1 was found to be lower in patients with RA (beta coef. −9 [95% CI −15 to −4] mg/dl, *p*=0.001) and the difference in HDL cholesterol serum levels between populations was lost. Remarkably, ANGPTL4 and ApoC3 remain significantly upregulated and LPL decreased in patients with RA compared to controls.

**Table 2:** Multivariable analysis of the differences in lipid profile and angiopoietin-like protein 4, apolipoprotein C3, and lipoprotein lipase serum levels between RA patients and controls

	Controls (*n*=246)	RA patients (*n*=323)	Univariable model	Model #1 beta coef. (95% CI), *p*	Model #2 beta coef. (95% CI), *p*
Lipid profile			*p*		
Cholesterol, mg/dl	198 ± 45	203 ± 38	0.12	5 (−2–12), 0.18	**14 (6**–**23), 0.001**
Triglycerides, mg/dl	144 ± 68	149 ± 88	0.44		
HDL cholesterol, mg/dl	52 ± 15	56 ± 15	**0.001**	1 (−1–4), 0.29	
LDL cholesterol, mg/dl	117 ± 37	117 ± 33	0.96		
LDL:HDL cholesterol ratio	2.38 ± 0.89	2.25 ± 0.94	0.10	0.01 (−0.15–0.17), 0.95	
Non-HDL cholesterol, mg/dl	146 ± 40	147 ± 38	0.72		
Lipoprotein (a), mg/dl	38 (14–101)	33 (11–111)	0.99		
Apolipoprotein A1, mg/dl	174 ± 39	170 ± 29	0.12	**−9 (−15 to −4), 0.001**	**−9 (−16 to −3), 0.006**
Apolipoprotein B, mg/dl	104 ± 29	107 ± 47	0.38		
Apo B:Apo A ratio	0.61 ± 0.18	0.64 ± 0.25	0.097	**0.05 (0.01–0.09), 0.008**	**–**
Atherogenic index	4.01 ± 1.12	3.88 ± 1.33	0.22		
Angiopoietin-like protein 4, ng/ml	73 (47–121)	151 (90–290)	**<0.001**	**215 (147–284), <0.001**	**295 (213–376), <0.001**
Apolipoprotein C3, mg/dl	6.2 ± 5.6	8.8 ± 5.2	**<0.001**	**2.9 (1.9–3.9), <0.001**	**2.9 (1.7–4.0), <0.001**
Lipoprotein lipase, ng/ml	230 (183–328)	99 (60–156)	**<0.001**	**−121 (−160 to −82), <0.001**	**−174 (−213 to −135), <0.001**

Data represent means ± standard deviation or median (interquartile range) when data were not normally distributed

*HDL* high-density lipoprotein, *LDL* low-density lipoprotein

Model #1: adjusted for sex, body mass index, abdominal circumference, hypertension, diabetes, C-reactive protein, and statins (variables with a *p* value < 20 difference between patients and controls)

Model #2: adjusted for model #1 + rest of lipid molecules (with a *p* value < 0.20 in the univariate analysis) other than the one that is compared

Because collinearity LDL cholesterol, LDL:HDL ratio, non-HDL cholesterol, apoB:apoA, and atherogenic index were excluded of the multivariable analyses in model 2

Because lipid-related molecules are interrelated (they share metabolic pathways and it is not easy to separate the effect of one from the others), we performed a multivariable analysis adjusting for demographics and CV risk factors plus all the lipid-related molecules that were found to be different between patients and controls in the univariable analysis (model 2 in Table 2). Because of collinearity, lipid molecules derived from a formula were excluded from the regression models (LDL cholesterol, LDL:HDL ratio, non-HDL cholesterol, apoB:apoA, and atherogenic index). In this final multivariable model, ANGPTL4, ApoC3, and LPL were found to be different in RA patients compared to controls: ANGPTL4 and ApoC3 were found to be higher and LPL lower.

**Fig. 1:**
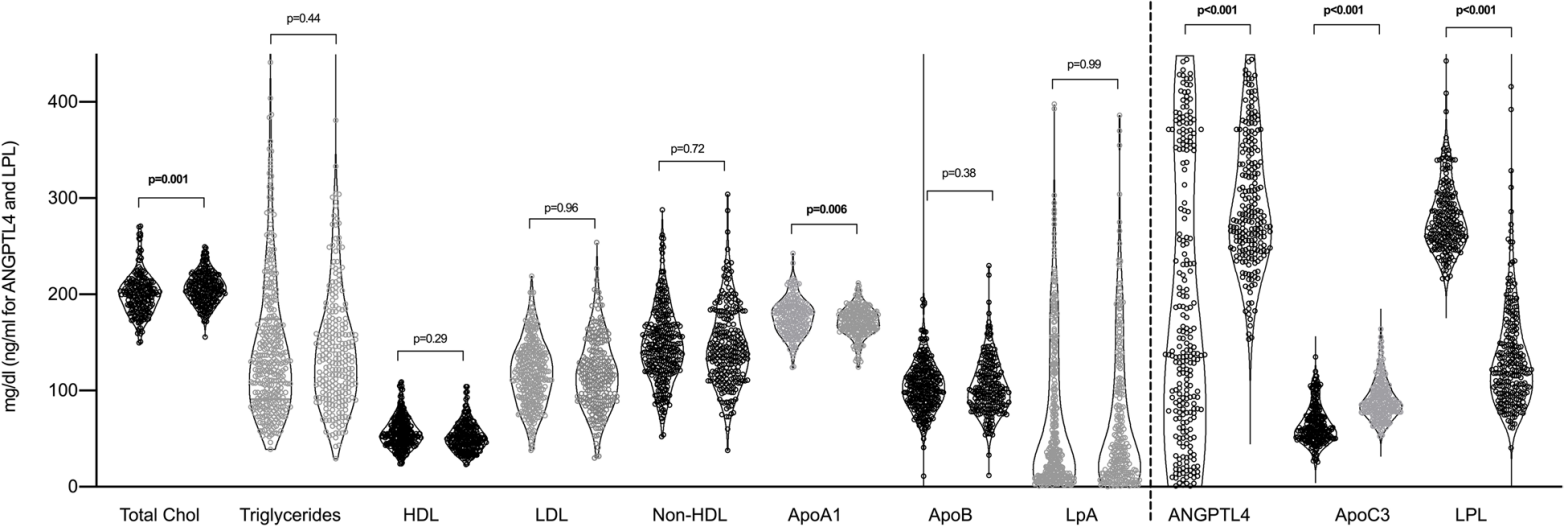
Multivariable differences in main lipid profile molecules between controls and patients with rheumatoid arthritis. For each molecule, left violin plot represents controls, and right violin plot refers to patients

Figure 1 shows a graphical representation of the differences in main lipid molecules between patients and controls.

### Disease-related data relation with angiopoietin-like protein 4, apolipoprotein C3, and lipoprotein lipase axis

Disease activity scores were not related to the ANGPTL4, ApoC3, and LPL molecules (Table 3). However, some associations were found with acute phase reactants. In this sense, the ESR was negatively correlated with ANGPTL4 (beta coef. −3 [95% CI −6 to −1] ng/ml, 0.020) but positively correlated with LPL (beta coef. 1 [95% CI 0–3] ng/ml, *p*=0.033). Besides, CRP was positively and significantly associated with ApoC3 (beta coef. 0.05 [95% CI 0.01–0.10] mg/dl, *p*=0.013). Similarly, patients on hydroxychloroquine, sulfasalazine, and anti-TNF therapies showed higher and significant serum levels of LPL (Table 3).

**Table 3:** Disease-related data relation with angiopoietin-like protein 4, apolipoprotein C3, and lipoprotein lipase axis

	ANGPTL4, ng/ml	Apo C3, mg/dl	LPL, ng/ml
	beta coef. (95% CI), *p*		
Disease-related data			
Disease duration, years	−7 (−14–0), 0.058	0.04 (−0.04–0.12), 0.32	4 (0–7), 0.025
CRP, mg/l	1 (−3–5), 0.67	**0.05 (0.01–0.10), 0.013**	1 (−1–3), 0.46
ESR, mm/1st hour	−**3 (**−**6 to** −**1), 0.020**	−0.00 (−0.03–0.03), 0.93	**1 (0–3), 0.033**
Rheumatoid factor	18 (−116–153), 0.79	0.2 (−1.2–1.6), 0.77	35 (−28–99), 0.27
ACPA	117 (−12–247), 0.075	0.2 (−1.2–1.5), 0.80	61 (−5–128), 0.068
DAS28-ESR	−19 (−93–16), 0.17	−0.4 (−1.0–0.2), 0.18	7 (−18–32), 0.58
DAS28-PCR	−15 (−75–45), 0.63	−0.4 (−1.0–0.3), 0.25	2 (−26–30), 0.88
SDAI	0 (−3–4), 0.88	0.03 (−0.01–0.06), 0.17	0 (−1–2), 0.70
CDAI	−2 (−10–6), 0.66	−0.08 (−0.16–0.01), 0.069	0 (−3–4), 0.90
HAQ	−25 (−128–77), 0.62	−0.3 (−1.4–0.8), 0.60	**52 (9**–**94), 0.017**
Current drugs			
Prednisone	22 (−105–149), 0.73	−0.1 (−1.4–1.3), 0.94	−10 (−69–49), 0.74
Prednisone, mg/day	16 (−28–59), 0.47	−0.2 (−0.5–0.1), 0.15	1 (−6–7), 0.87
NSAIDs	21 (−101–144), 0.73	−0.4 (−1.7–0.8), 0.50	9 (−48–66), 0.77
DMARDs	−20 (−181–140), 0.80	−0.4 (−2.1–1.3), 0.63	29 (−53–110), 0.49
Methotrexate	25 (−105–155), 0.71	0.4 (−0.9–1.8), 0.53	41 (−24–106), 0.21
Leflunomide	−50 (−218–118), 0.56	0.4 (−1.4–2.2), 0.67	51 (−17–119), 0.14
Hydroxychloroquine	257 (−229–744), 0.30	−3.5 (−8.7–1.6), 0.18	**116 (30**–**202), 0.008**
Salazopyrin	150 (−819–1120), 0.76	−3.6 (−13.9–6.6), 0.49	**143 (41**–**245), 0.006**
Anti-TNF therapy	−11 (−182–159), 0.90	−0.2 (−2.1–1.6), 0.79	**101 (33**–**169), 0.004**
Tocilizumab	53 (−204–311), 0.68	−1.3 (−4.0–1.4), 0.35	9 (−118–137), 0.89
Rituximab	−202 (−601–197), 0.32	0.0 (−4.3–4.2), 0.99	−23 (−228–181), 0.82
Abatacept	−182 (−581–217), 0.37	3.9 (−0.3–8.1), 0.069	−58 (−263–146), 0.58
JAK inhibitors	347 (−22–715), 0.065	−3.6 (−7.5–0.3), 0.072	−39 (−216–139), 0.67
History of extraarticular manifestations	−136 (−349–76), 0.21	0.4 (−1.9–2.7), 0.76	37 (−54–127), 0.42
Erosions	17 (−122–157), 0.81	−1.0 (−2.4–0.4), 0.16	57 (−7–121), 0.081

*NSAID* nonsteroidal anti-inflammatory drugs, *DMARD* disease-modifying antirheumatic drug, *TNF* tumor necrosis factor, Obesity, *ESR* erythrocyte sedimentation rate, *DAS28* Disease Activity Score in 28 joints, *CRP* C-reactive protein, *ACPA* anti-citrullinated protein antibodies, *CDAI* Clinical Disease Activity Index, *SDAI* Simple Disease Activity Index, *HAQ* Health Assessment Questionnaire

### Relationship of angiopoietin-like protein 4, apolipoprotein C3, and the lipoprotein lipase axis with subclinical atherosclerosis in patients with RA

Age, male gender, traditional CV risk factors, the use of statins, and some lipid profile-related molecules like triglycerides, LDL cholesterol, and lipoprotein (a) were significantly associated with the presence of carotid plaque or a higher cIMT (Table 4). Although ANGPTL4 and LPL were not related with carotid plaque, ApoC3 was associated with the presence of carotid plaque in patients with RA in the univariable analysis. However, this association was lost after multivariable analysis.

**Table 4:** Relation of angiopoietin-like protein 4, apolipoprotein C3, and lipoprotein lipase axis to subclinical atherosclerosis in RA patients

	Carotid plaque					cIMT, microns	
	Univariable			Adjusted		Univariable	Adjusted
	No=199	Yes=124	*p*	OR (95% CI)	*p*	beta coef. (95% CI), *p*	
Age, years	51 ± 10	61 ± 8	**<0.001**			**7 (6–8), <0.001**	
Female, *n* (%)	175 (88)	88 (71)	**<0.001**			**−88 (−126 to –50), <0.001**	
BMI, kg/m^2^	28 ± 5	28 ± 5	0.51			2 (−8–5), 0.14	
Abdominal circumference, cm	97 ± 14	97 ± 11	0.71			**2 (1–3), 0.004**	
Cardiovascular data							
CV risk factors, *n* (%)							
Current smoker	37 (19)	27 (22)	0.49			4 (−34–42), 0.84	
Obesity	67 (34)	37 (30)	0.47			21 (−10–54), 0.18	
Hypertension	49 (25)	52 (42)	**0.001**			**61 (29–93), <0.001**	
Diabetes mellitus	17 (9)	25 (20)	**0.003**			**87 (43–132), <0.001**	
Blood pressure, mm Hg							
Systolic	130 ± 18	138 ± 19	**0.001**			**2 (1–3), <0.001**	
Diastolic	81 ± 12	83 ± 11	0.077			1 (0.3), 0.061	
Statins, *n* (%)	46 (23)	57 (46)	**<0.001**			**51 (20–83), 0.002**	
Lipid profile							
Cholesterol, mg/dl	205 ± 37	201 ± 39	0.42			0.2 (−0.2–0.6), 0.24	
Triglycerides, mg/dl	138 ± 82	168 ± 93	**0.003**			0.1 (−0.0–0.3), 0.13	
HDL cholesterol, mg/dl	57 ± 15	55 ± 16	0.50			−0.7 (−1.7–0.3), 0.17	
LDL cholesterol, mg/dl	121 ± 32	112 ± 35	**0.030**			0.3 (−0.2–0.7), 0.24	
LDL:HDL cholesterol ratio	2.30 ± 0.97	2.18 ± 0.90	0.24			12 (−4–28), 0.14	
Non-HDL cholesterol, mg/dl	148 ± 39	146 ± 38	0.61			0.3 (−0.0–0.7), 0.085	
Lipoprotein (a), mg/dl	29 (10–87)	38 (14–132)	**0.018**			0.0 (−0.2–0.2), 0.74	
Apolipoprotein A1, mg/dl	170 ± 29	171 ± 30	0.70			0.0 (−0.5–0.5), 0.98	
Apolipoprotein B, mg/dl	108 ± 57	104 ± 24	0.51			0.2 (−0.2–0.5), 0.35	
Apo B:Apo A ratio	0.65 ± 0.28	0.63 ± 0.17	0.42			36 (−25–97), 0.25	
Atherogenic index	3.87 ± 1.38	3.89 ± 1.25	0.92			9 (−3–20), 0.13	
Axis							
Angiopoietin-like protein 4, ng/ml	143 (85–281)	161 (104–451)	0.15	1.00 (0.99–1.00)	0.14	**0.07 (0.03–0.10), <0.001**	**0.05 (0.02–0.08), <0.001**
Apolipoprotein C3, mg/dl	8.3 ± 4.7	9.8 ± 5.9	**0.028**	1.03 (0.97–1.09)	0.41	2.9 (−0.3–6.2), 0.077	0.4 (−2.6–3.4), 0.78
Lipoprotein lipase, ng/ml	91 (53–149)	108 (76–166)	0.34			0.03 (−0.03–0.09), 0.26	0.01 (−0.04–0.6), 0.73

*BMI* body mass index, *CV* cardiovascular, *HDL* high-density lipoprotein, *LDL* low-density lipoprotein, *OR* odds ratio, *cIMT* carotid intima-media thickness

Carotid plaque and cIMT are dependent variables. Multivariable logistic regression—odds ratios—is adjusted for age, gender, hypertension, diabetes mellitus, statins, triglycerides, LDL cholesterol and lipoprotein (a). Multivariable linear regression is adjusted for age, gender, abdominal circumference, hypertension, diabetes mellitus, statins, triglycerides, and HDL cholesterol

Regarding cIMT, ApoC3 and LPL were not associated with cIMT. Contrary, a significant association between ANGPTL4 and cIMT was found after full multivariable regression analysis (beta coef. 0.05 [95% CI 0.02–0.08] microns, *p*<0.001) (Table 4).

### Mediation analysis and hypothetical pathways of disruption of the axis

**Fig. 2:**
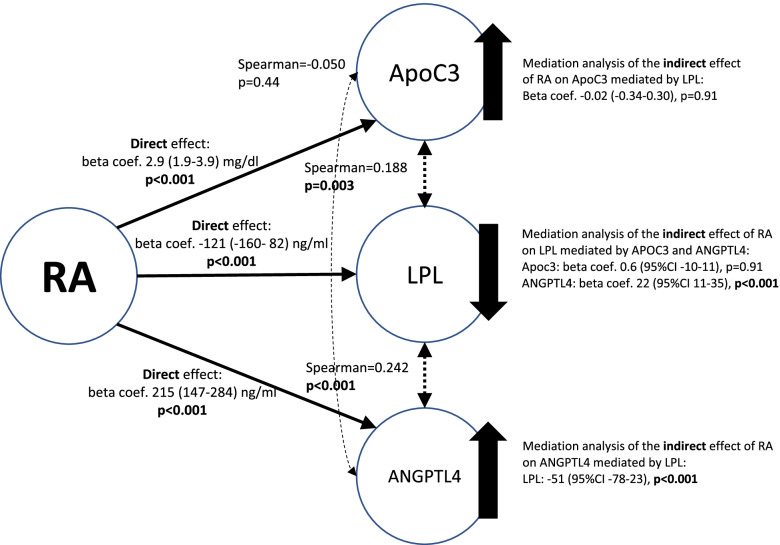
Hypothetical pathways of the disruption of the ANGPTL4, ApoC3, and LPL axis. Direct effects of RA on the three molecules are shown in continued arrows. Correlation between them is illustrated in discontinued arrows. Mediation analysis of the effect of RA on every molecules mediated by the others is shown. Since ApoC3 and ANGPTL4 did not correlate, mediation analysis of one over the other does not apply

As previously mentioned, patients with RA had higher serum levels of ANGPTL4 and ApoC3 but lower circulating LPL (Fig. 2). ApoC3 and LPL (Rho Spearman=0.188, *p*=0.003) and ANGPTL4 and LPL (Rho Spearman=0.242, *p*<0.001) serum levels correlated between them (discontinued arrows). This was not the case of the correlation between ApoC3 and ANGPTL4, which did not show a statistical significance (Rho Spearman=−0.050, *p*=0.44). Figure 2 shows a hypothetical representation of the relationship of these molecules.

As we were interested in evaluating whether the disturbance of the axis molecules was interrelated, or the disruption of one molecule was the consequence of the modification of another, we performed a mediation analysis. In this sense, direct and indirect effects of RA over each molecule were analyzed studying the mediation effect of the other two. Since ApoC3 and ANGPTL4 were not correlated, the study of the mediation effect of RA on each of them mediated for the other did not apply. The mediation analyses of the indirect effect of RA on ApoC3 mediated by LPL and on LPL mediated by ApoC3 were not significant (Fig. 2). In contrast, the indirect effects of RA on ANGPTL4 mediated by LPL and on LPL mediated by ANGPTL4 were significant but small in size. Furthermore, the analysis of the direct effect of RA over the three molecules remain statistically significant when mediation analysis variables were included in the multivariable models (Fig. 2). All the mediation analyses were evaluated adjusting for the same variables of model 1 of Table 2.

## Discussion

Our study is the first to analyze the key molecules related to triglyceride metabolism in RA. According to our results, the axis constituted by ANGPTL4, ApoC3, and LPL is disrupted in RA and related to subclinical CV disease in these patients. Our approach focuses on how the inflammatory state produced by RA modifies the lipid profile and how, in turn, it can influence the atherosclerotic burden observed in patients with RA.

In the current work, most of the lipid profile molecules did not differ between patients and controls. This means that inflammatory dyslipidemia, which has been described in patients with RA [7], was not present in our cohort. It may be because most of the patients recruited in our study had low or moderate disease activity. This reinforces our hypothesis, since, for this reason, the disruption in the ANGPTL4, ApoC3, and LPL axis found in our study cannot be attributed to differences in the lipid profile between patients and controls. In this regard, such a disruption of the ANGPTL4-LPL-ApoC3 axis in RA patients compared to controls was found to be significant after a fully multivariable analysis that included traditional CV risk factors and other lipid molecules.

The positive regulation of ApoC3 in RA found in our study has been previously described. Regarding this, a study in 94 RA patients and 79 controls showed that the serum concentration of ApoC3 was found to be higher in patients compared to controls [19]. This is of potential relevance since ApoC3 has been recognized as a link between atherogenic and inflammatory processes not only in the general population [20] but also in subjects with RA. In this sense, in a study of 152 patients with RA who had a coronary artery calcium score evaluated at baseline and at year 3, ApoC3 was found to be significantly elevated in progressors compared to non-progressors [21]. In our study, ApoC3 was associated with carotid plaque and cIMT in the univariable analysis, but this association did not reach statistical significance after multivariable adjustment.

In a small study of 17 women with RA and 16 age- and sex-matched controls, LPL mass and activity levels were significantly lower in RA patients [22]. This is consistent with our study, which also found a decrease in LPL. Moreover, in our work, we found a positive relationship between LPL and ESR but not with disease activity scores. However, the RA patients in our series showed reduced LPL levels after multivariate adjustment, reinforcing the claim that the disease itself may be responsible for the LPL decrease.

ANGPTL4 was positively associated with cIMT in our work which is consistent with previous studies that showed a link between ANGPTL4 serum levels and atherosclerosis. In this regard, serum ANGPTL4 levels in a series of 712 patients with stroke due to large artery atherosclerosis disease were significantly higher than those in 828 controls after adjustment for other risk factors [23]. Pathological studies indicate that cIMT mainly represents hypertensive medial hypertrophy or thickening of smooth muscles in the media. In contrast, carotid plaques probably represent a later stage of atherogenesis related to inflammation, endothelial dysfunction, oxidative stress, and smooth muscle cell proliferation [24]. Therefore, since cIMT is biologically distinct from plaque and represents a different process, it is possible that in RA patients ANGPTL4 may have more influence on cIMT than on plaque development.

Given that the three axis molecules evaluated in our study are interrelated and mutually modified, we performed a mediation analysis to clarify whether the effect of the disease in each of them was mediated by the alteration of the others. However, it did not yield statistically significant results or, when significant, the indirect (mediated) effect was small. This means that the modification in the three molecules may be because of the disease itself and does not seem to be produced from the modification that each of the three molecules can exert on the others.

We recognize as a potential limitation of our study that we measured LPL serum levels and not its enzymatic activity. However, although serum LPL is catalytically inactive, its mass reflects the level of systemic LPL biosynthesis and there is an excellent correlation between mass and LPL activity as reported elsewhere [25].

## Conclusion

In conclusion, the axis related to triglyceride metabolism constituted by ANGPTL4, ApoC3, and LPL is different in patients with RA and healthy controls. Since the serum levels of ANGPTL4 are related to cIMT, this molecule may represent a biomarker of subclinical atherosclerosis in these patients. Our findings may contribute to improving the understanding of the relationship between inflammatory dyslipidemia and CV disease in patients with RA.

## Data Availability

The data sets used and/or analyzed in the present study are available from the corresponding author upon request.

## Abbreviations

ACPA: Anti-citrullinated peptide/protein antibody
ANGPTL4: Angiopoietin-like protein 4
ApoC3: Apolipoprotein C-3
BMI: Body mass index
CDAI: Clinical Disease Activity Index
CI: Confidence of interval
cIMT: Carotid intima-media thickness
CRP: C-reactive protein
CV: Cardiovascular
DAS28: Disease Activity Score in 28 joints
DMARD: Disease-modifying antirheumatic drug
ELISA: Enzyme-linked immunosorbent assay
ESR: Erythrocyte sedimentation rate
HAQ: Health Assessment Questionnaire
HDL: High-density lipoprotein
IQR: Interquartile range
LDL: Low-density lipoprotein
LPL: Lipoprotein lipase
NSAID: Nonsteroidal anti-inflammatory drug
OR: Odds ratio
RA: Rheumatoid arthritis
SD: Standard deviation
SDAI: Simplified Disease Activity Index
TNF-α: Tumor necrosis factor-α
